# Aminoglycosides: From Antibiotics to Building Blocks for the Synthesis and Development of Gene Delivery Vehicles †

**DOI:** 10.3390/antibiotics9080504

**Published:** 2020-08-11

**Authors:** Maria Cristina Bellucci, Alessandro Volonterio

**Affiliations:** 1 Department of Food, Environmental and Nutritional Sciences, Università degli Studi di Milano, via Celoria 2, 20133 Milano, Italy; cristina.bellucci@unimi.it; 2 Department of Chemistry, Material and Chemical Engineer “Giulio Natta”, Politecnico di Milano, via Mancinelli 7, 20131 Milano, Italy

**Keywords:** aminoglycosides, gene delivery, non-viral vectors

## Abstract

Aminoglycosides are a class of naturally occurring and semi synthetic antibiotics that have been used for a long time in fighting bacterial infections. Due to acquired antibiotic resistance and inherent toxicity, aminoglycosides have experienced a decrease in interest over time. However, in the last decade, we are seeing a renaissance of aminoglycosides thanks to a better understanding of their chemistry and mode of action, which had led to new trends of application. The purpose of this comprehensive review is to highlight one of these new fields of application: the use of aminoglycosides as building blocks for the development of liposomal and polymeric vectors for gene delivery. The design, synthetic strategies, ability to condensate the genetic material, the efficiency in transfection, and cytotoxicity as well as when available, the antibacterial activity of aminoglycoside-based cationic lipids and polymers are covered and critically analyzed.

## 1. Introduction

“The future of aminoglycosides: the end or renaissance?”. This is the title of an excellent review by Prof. Sylvie Garneau-Tsodikova et al. published exactly ten years ago [1]. In this paper, the authors interrogated the future of aminoglycosides (AGs) as antibiotics. Indeed, AGs, displacing broad-spectrum activity against both Gram-negative and Gram-positive bacteria, have been used to fight pathogenic bacteria for a long time, actually since 1943 when streptomycin was discovered [2]. The mode of action of AGs has been widely investigated, showing that AGs are able to bind to the decoding A-site of the 16S ribosomal RNA of bacteria, resulting in the interference of protein synthesis that eventually leads to cell death and to helix 69 in the 50S ribosomal subunit hampering mRNA/tRNA translocation and ribosome recycling [3,4,5]. These recognition events depend mainly on electrostatic interactions between the positively charged ammonium groups on the AG scaffold and the negatively charged phosphates present on the target, and, secondary, on less important contributions coming from hydrogen bond networking. However, the heavy use (or abuse) of AGs during the time resulted in the development of antibiotic resistance through different mechanisms encompassing reduced uptake and increased efflux, modification of the ribosomal target, and AG regioselective modification (namely acetylation, phosphorylation, and nucleoside transferring) by AG-modifying enzymes [6]. Moreover, the clinical efficiency of AGs has been also hindered by their nephrotoxicity [7] and ototoxicity [8]. In order to fight the acquired resistance and/or toxicity, different strategies for the selective functionalization of AGs have been developed and recently reviewed [9,10]. However, although many structure–activity relationship (SAR) studies have been addressed, resistance against different microorganisms as well as inherent toxicity remains a big challenge to be faced. Ten years later from the above-mentioned review, the research on the AGs is still ongoing, and although the failure of different projects could have elicited the end of AGs, the better understanding of the mode of action and chemistry, which triggered an extension of their applications, has led to a renaissance of these drugs. Indeed, AGs are currently being investigated not only as building blocks for the synthesis of new antibacterial agents, but also as potential antifungals, antiprotozoals, genetic regulating agents, and as cationic heads in the development of multifunctional drug delivery vectors [11].

In this review, we will focus the reader’s attention on the use of AGs as scaffolds for building molecular or polymeric vehicles to facilitate the introduction of genes into cells (gene delivery). After a brief introduction on AG classification and the gene delivery field, we will provide an overall description of AG-based cationic lipids and polymers projected as gene delivery vectors, encompassing the chemistry, the ability to condensate DNA/RNA, the efficiency on delivering the genetic material to the target cell organelles, cytotoxicity, and, when available, the antibacterial activity. Although a certain amount of work has been done in this field, a real definition of which parameters should be taken into consideration to help in the design of new AG-based liposomes and/or polymers for efficient gene delivery application is still missing. The scope of this review is to collect, for the first time, all the information available on the literature and to try to define a sort of “rule of thumb”, which will be useful for the future development of more efficient AG-based gene delivery vectors. We also believe that this “relatively unexplored” field of application of AG antibiotics could further help in the future of the renaissance of this class of fascinating natural molecules.

## 2. Classification and Chemistry of Aminoglycosides (AGs)

**Figure 1:**
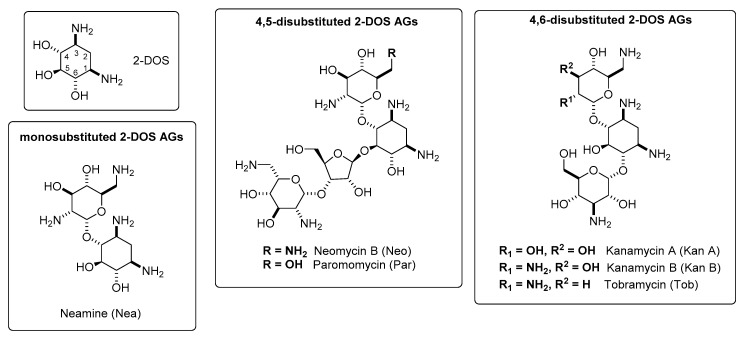
Structures of some aminoglycosides (AGs) discussed in this review.

AGs are a class of naturally occurring and semi-synthetic polyamino sugars that can be classified based on the substitution of the 2-deoxystreptamine (2-DOS) ring, a common building block present on most AGs (Figure 1). Accordingly, there are three main classes of AGs: 4,5-disubstituted 2-DOS AGs; 4,6-disubstituted 2-DOS AGs; and a third class encompassing monosubstituted 2-DOS AGs and streptomycin.

**Figure 2:**
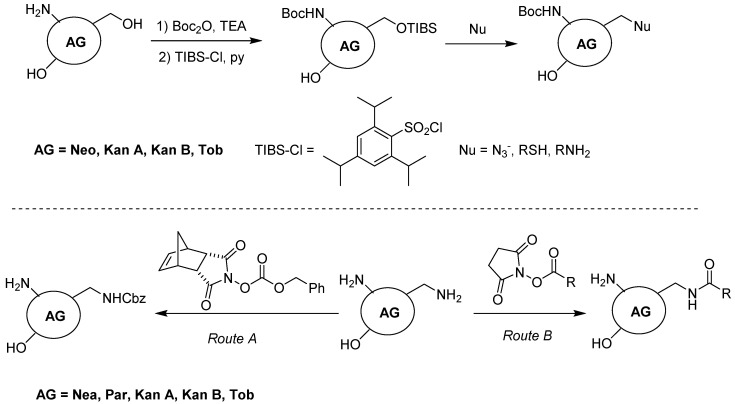
General strategies for the chemoselective functionalization of AGs.

At first sight, the selective functionalization of AGs could seem an insurmountable task due to the presence of many hydroxy and amino functional groups, each one having similar reactivity. However, the extensive work done on the search of AG derivatives in order to overcome antibiotic resistance and/or toxicity has paved the way to solving this challenging task. In general, some functional groups are much more available to functionalization being more accessible because they are less sterically hindered. More specifically, some AGs such as Neo, Kan A and B, and Tob bear only one primary hydroxy group that can be exploited for selective functionalization (Figure 2). Actually, after the protection of the amino functions with Boc or Cbz, this primary hydroxy group is selectively transformed in a good leaving group by reaction with an excess of sterically hindered triisopropylbenzensulfonyl chloride (TIBS-Cl). The resulting sulfate is displaced by different nucleophiles such as amines, azide, and thiols, in order to obtain the final adducts or intermediates ready for further chemoselective functionalization. Analogously, the presence of only one, less sterically hindered methyleneamino function in other AGs such as Nea, Par, Kan A and B, and Tob could be exploited to discern the reactivity of the amino functional groups. Accordingly, by reacting Nea, Par, Kan A and B, and Tob with Cbz-*N*-hydroxy-5-norbornene-2,3-dicarboxylic acid imide, or more in general, with *N*-hydroxysuccinimide (NHS) esters, chemoselective protection or acylation of the methyleneamino moiety can be achieved respectively. However, the latter strategy is less pursued, mainly for two reasons: (1) the yields are not very high, and (2) one amino functional group is sacrificed, the amino groups being generally important for the function of AGs. Along with the former strategies, other synthetic pathways relaying on multistep protection/functionalization/deprotection approaches have been developed. However, since the chemistry exploited for the synthesis of AG-based gene delivery vectors reviewed herein pertains to the strategies depicted in Figure 2, we will not go through these strategies, suggesting that interested readers refer to the comprehensive review cited in [9,10].

## 3. Gene Delivery

**Figure 3:**
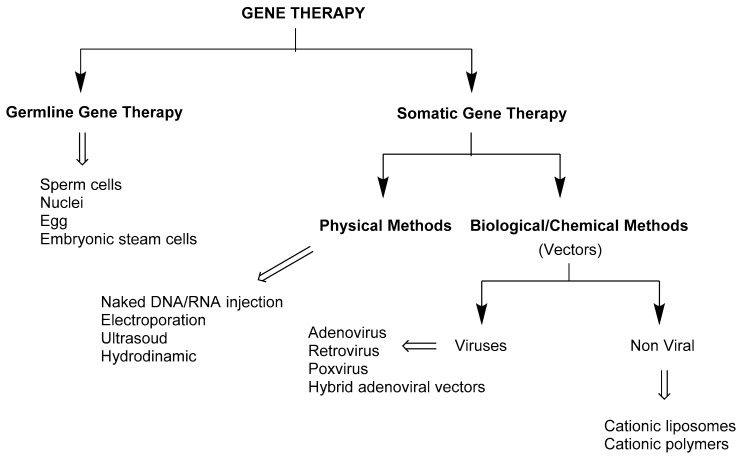
Different gene delivery systems.

Gene delivery is a fundamental step of gene therapy that wants to prevent or treat not only genetic diseases, but also other disorders including vascular diseases, neurodegenerative and infectious disorders, and many types of cancers by introducing genetic material into specific cells in order to replace a defective or missing gene, to inactivate a gene that is functioning improperly, or to add a gene/protein function [12,13,14]. There exist two major categories of gene therapy, encompassing germline and somatic gene therapy (Figure 3) [15]. While germline gene therapy, although very promising, suffers from ethical concerns and is prohibited in many countries [16,17], somatic gene delivery systems have finally reached the clinical and market after facing quite numerous obstacles and failures [18]. Ex vivo somatic gene therapy relies on the manipulation in vitro of selected cells that are first explanted from the body and, after the manipulation, re-implanted into the target tissues. On the other hand, in in vivo somatic gene delivery, the delivering of the genetic material occurs in situ [19]. However, two major barriers must be overcome for this technology to be efficient: both DNA and RNA must first be protected from the action of hydrolyzing enzymes present in the extracellular matrix and, second, must be helped to cross the cell membrane since they are not able to enter the cell spontaneously mainly due to electrostatic repulsion with glycoproteins and phospholipids. In order to surmount such barriers, many physical methods have been developed, encompassing, among others, mechanical, electrical, hydrodynamic, and ultrasonic [20]. Another way to achieve these goals is to develop a biological or chemical helper, referred to as a vector, that should be able to pack and protect the gene outside the cell, cross the cell membrane, and finally deliver the gene into the target organelle. Such vehicles are classified as viral and non-viral gene delivery vectors [21,22]. Viruses are so far the most efficient gene delivery vectors, having reached in some cases clinical approval by the U.S. Food and Drug Administration (FDA) and the market [23]. However, the use of viruses is hampered by their marked immunogenicity, limited carrying capacity, and insertional mutagenesis. In order to overcome the drawbacks associated with the use of viral vectors, scientists are focusing attention on developing efficient and non-cytotoxic non-viral vectors, which are generally cationic liposomes/micelles or polymers that are able to condensate the genetic material through electrostatic interactions. Liposomes are composed of amphiphilic molecules substantially made of a cationic head and lipophilic tail(s) tethered by a linker [24]. The cationic head is important for the condensation of the genetic material through electrostatic interactions, the number of cationic charges, the nature, and tridimensional disposition of the cationic head groups having a great influence on the transfection efficiency [25]. Lipophilic tails can be classified as linear hydrocarbon alkyl chains, encompassing saturated and unsaturated chains, (per)fluorinated alkyl chains, and cholesterol derivatives. These are important for the stability of the liposomes, membrane crossing, and influence the transfection efficiency [26]. Finally, the linker, although probably less considered if not for chemical purposes, is important because its length, tridimensional geometry, and chemical composition might affect the transfection efficiency, biodegradability, and cytotoxicity of the vectors [27].

There is a great variety of polymeric gene delivery vectors, which renders their classification quite difficult. Polymers used in gene delivery encompass linear, branched, or dendrimeric structures and their monomers range from simple molecules such as ethyleneamine, ethylenamidamine, and acrylate to more sophisticated carbohydrates, amino acids, carbosilane, and phosphorous containing monomers [28]. Natural polymers such as chitosan or commercially available polymers like linear and branched polyethyleimine (lPEI or bPEI) or polyethylenaminoamine (PAMAM) dendrimers of different generations are probably the most widely used polymers in this field, with bPEI 25 KDa considered the gold standard [29]. Polymeric cationic gene delivery vectors often suffer from the transfection efficiency/cytotoxicity paradox: the more cationic moieties in the structure, the more efficient and the more cytotoxic the polymer. For this reason, as well as for their polyplex instability, poor selectivity, insufficient endosomal escape, and difficult DNA release, there is great interest in the synthesis of new polymers and in the functionalization of non-viral polymeric vectors with different moieties, encompassing lipidic chains, cationic moieties, amino acids, peptides, and saccharides [30]. In this review, we will cover the use of AGs as building-blocks for the synthesis of liposomes, for the functionalization of commercially available polymers, and for the synthesis of hyperbranched polymers.

## 4. AG-Based Lipid Gene Delivery Vectors

In the quest for efficient and safe non-viral gene delivery vectors, cationic lipids are very attractive because they are relatively easy to design, synthesize, and characterize, being made of tethering three modules: a cationic moiety, a lipophilic tail(s), and a linker to connect them. Among the cationic moieties, AGs have reached a certain interest in the last twenty years. The rational beyond the use of AGs is manifold: (1) they are natural binders of a great variety of RNAs through electrostatic interactions [31]; (2) although they have lower affinity for DNA, some AGs are able to tightly interact with DNA and to protect it from nucleases [32]; (3) they possess a broad spectrum antibacterial activity that could be exploited for the preparation of multifunctional vectors [1,6]; (4) there is a good variety of commercial available AGs with a different number of amines with different spatial dispositions that could be exploited to fine tune the efficiency/cytotoxicity of the corresponding liposomes; and (5) the chemistry of AGs is well documented [9,10]. Aside from the AG moiety, the performances of these vectors strongly depend on the lipophilic tail(s) tethered and on the nucleic acid delivered. For this reason, we decided to organize this section in different sub-sections describing the behavior of AG-based lipidic vectors built with “conventional lipidic tails” such as cholesterol or linear hydrocarbon chains for DNA delivery and RNAs delivery. A third section will be dedicated to AG-based lipidic vectors bearing “non-conventional” lipidic tails.

### 4.1. AG-Based Lipid Vectors for DNA Delivery

**Figure 4:**
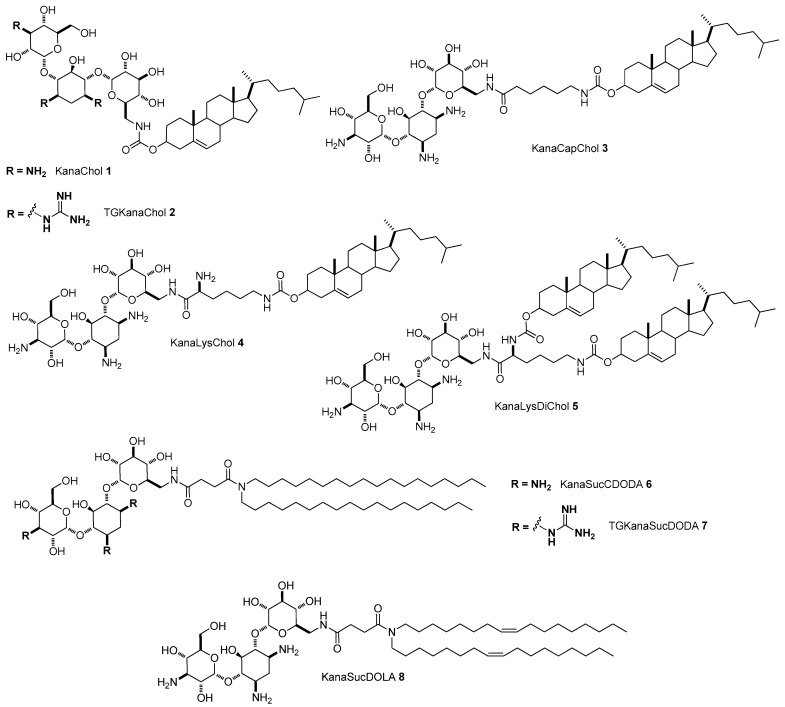
Structures of Kanamycin A-based lipids **1–8**.

The first paper dealing with the use of AGs to build gene delivery vectors appeared in 2002 where the synthesis of two kanamycin-cholesterol conjugates, namely kanamycin-cholesterol KanaChol **1** and the corresponding triguanidinium-kanamycin-cholesterol TGKanaChol **2**, was described (Figure 4) [33].

Kanamycin A was tethered to cholesterol via a carbamoyl bond, a functional group that is considered both chemically stable and biodegradable. According to route A depicted in Figure 2, thus sacrificing one out of the four amino groups on the kanamycin scaffold, the unique reactivity of the only methyleneammino group has been exploited [34]. Liposomes made with KanaChol **1** and TGKanaChol **2** in Hepes buffer solution (pH 7.4) were able to deliver in vitro plasmid pCMV-Luc, a luciferase-expressing plasmid DNA, in an efficient way, showing almost the same results both in terms of efficiency and in terms of cytotoxicity, whereas kanamycin itself was ineffective. In more detail, the transfection in three cell lines, namely HeLa, HEK293, and COS cell lines, was most successful at a *N*/*P* ratio (nitrogen to phosphate groups) around 5, resulting in a decreasing efficiency for higher value of *N*/*P* ratios, probably due to an increase of toxic effects. This result was quite unforeseen since TGKanaChol **2** was expected to be more efficient than KanaChol **1**. Indeed, guanidinoglycosides are known to have both higher RNA affinity and much greater ability to cross cell membranes compared to their unguanidinylated counterparts [35,36,37,38,39,40]. Interestingly, co-formulation with dioleoylphosphatidylthanolamine (DOPE), a neutral phospholipid that is known to help endosomal escape of the internalized lipoplexes [41], did not affect the in vitro transfection efficiency of KanaChol **1**, demonstrating that KanaChol **1** is an efficient non-viral gene delivery vector that does not need liposomal formulation. However, co-formulation with DOPE was useful in order to deliver cholesterol-poly(ethylene glycol) (Chol-PEG) stabilized DNA in vivo through intranasal administration into mouse airways.

In order to check the influence of the linker and lipophilic tails, a small library of kanamycin-based liposomes were synthetized using 6-aminocapronic acid (Cap), lysine (Lys), and succinic acid (Suc) linkers, and cholesterol, dioleyl amine, and disteraryl amine lipophilic tails (Figure 4) [42]. In more detail, following the same synthetic strategy (Path A, Figure 2) three new derivatives with cholesterol were prepared, namely KanaCapChol **3** having a 6-aminocapronic acid linker, and two derivatives having a lysine linker, one of them, KanaLysChol **4**, bearing one cholesterol, while the other, KanaLysDiChol **5**, bears two cholesterol units. Moreover, two derivatives with a lipophilic dioctadecylamine and succinic acid linker, namely KanaSucDODA **6** and the corresponding triguanidinylated TGKanaSucDODA **7**, and one bearing dioleylamine instead, KanaSucDOLA **8** has also been synthetized [42]. The new cholesterol derivatives **3**–**5** were not fully soluble in water, thus lipolexes were prepared in the presence of DOPE and tested at different *N*/*P* ratios on five mammalian cell lines. In four out of the five cell lines tested, new compounds showed better transfection efficiencies compared to KanaChol **1**, and the best efficiencies were reached at *N*/*P* ratios around 5 and 10, showing that the use of longer spacers between the cationic head group and the lipophilic moiety is favorable and provides better physico-chemical properties to the lipoplexes. Notably, the presence of a further amino function in KanaLysChol **4** compared to KanaCapChol **3** did not markedly affect the transfection efficiency. In addition, the substitution of cholesterol with aliphatic long chains such as in KanaSucDODA **6** and KanaSucDOLA **8** resulted in better efficiencies when administrated without DOPE, probably because the fluidity of the lipid layers benefited from this substitution. Co-formulation with DOPE produced a different effect, slightly increasing the efficiency of KanaSucDODA **6** and slightly decreasing that of KanaSucDOLA **8**. As already observed for KanaChol **1** and TGKanaChol **2**, guanidinylation of KanaSucDODA **6** did not enhance the transfection properties of the vector, TGKanaSucDADA **7**, being much less efficient than **6**. All these data confirmed that AG-based gene delivery vectors are efficient and non-cytotoxic gene delivery vectors when administrated at low values of the *N*/*P* ratio, as the vectors have a long spacer and long alkyl chains as lipophilic tails are much more powerful. However, the beneficial effect of the longer linker was not observed in vivo, since only KanaLysChol **4** resulted in being more efficient than KanaChol **1** when co-administrated with DOPE and Chol-PEG to BALB/c mice through intranasal administration.

**Figure 5:**
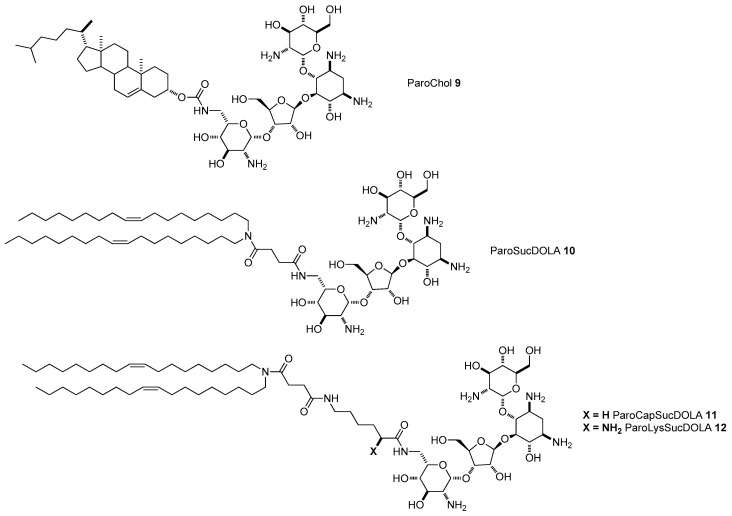
Structures of paromomycin-based lipids **9**–**12**.

**Figure 6:**
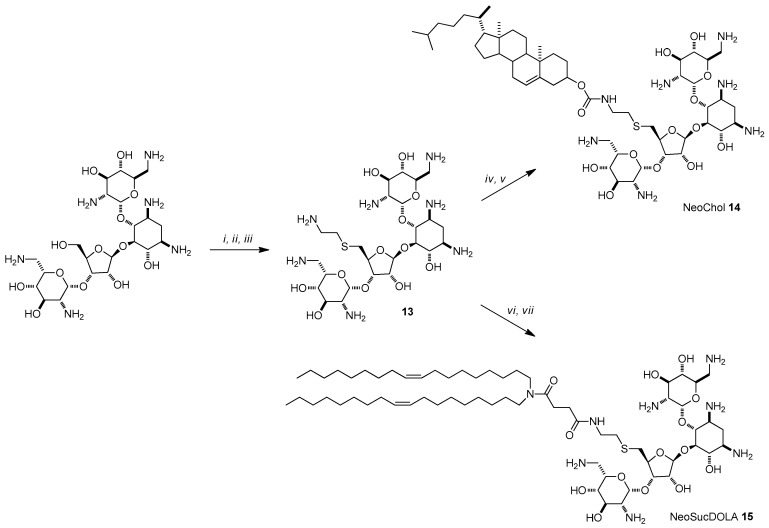
Synthesis of neomycin-based lipids **14**, **15**: (i) Boc_2_O, TEA, DMF/H_2_O, (ii) TIBS-Cl, pyridine, (iii) 2-aminoethanethiol, EtONa/EtOH, (iv) cholesteryl chloroformate, TEA, THF/DMF, (v) TFA/DCM, (vi) *N*-succinyl-dioleylamine, HOAt/EDC, DFM/DCM, (vii) TFA/DCM.

**Figure 7:**
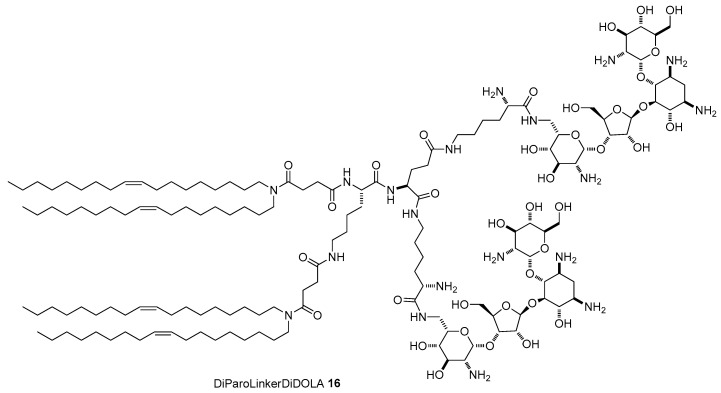
Structure of diparomomycin lipid **16**.

The impact of the cationic moiety of AG-based liposomes has been addressed through the synthesis of different derivatives starting from neomycin, paromomycin, and neamine. Following the synthetic strategy depicted in Path A of Figure 2, four paromomycin derivatives were prepared, namely ParoChol **9** (the analog of KanaChol **1** where paromomycin is linked to cholesterol through a carbamate moiety), ParoSucDOLA **10** (the analogue of KanaSucDOLA **8**), and two derivatives, ParoCapSucDOLA **11** and ParoLysSucDOLA **12**, bearing dioleyl lipidic chains and longer linkers made by coupling 6-aminocaproic acid or lysine to succinic acid, respectively (Figure 5) [43]. Path B of Figure 2 was exploited in order to synthetize the derivatives of neomycin B. Accordingly, after the protection of all the amino functional groups with ditertbutyldicarbonate, the only primary hydroxyl group was selectively functionalized as triisopropylbenzensulfate and substituted by thioethanolamine, giving rise to the formation of intermediate **13**, which was reacted with cholesterylchloroformiate and N-succinyl-dioleylamine, giving rise to the formation, after Boc deprotection, of liposomes NeoChol **14** and NeoSucDOLA **15**, respectively (Figure 6). Finally, in order to study the importance of the number of the amino moieties and the size of the cationic head group, dimeric paromomycin conjugate DiParoLinkerDiDOLA **17** was prepared using an ad hoc designed long glutamic acid-lysine-succinic acid linker (Figure 7).

The transfection efficiencies of these paromomycin- and neomycin-based liposomes have been evaluated at different values of *N*/*P* ratios (between 5 and 19) in two mammalian cell lines, namely HeLa and HEK293. All of these derivatives showed very good transfection efficiencies at their optimal charge ratio, with the dioleyl derivatives the best performing luciferase-expressing plasmid DNA vectors. Moreover, ParoSucDOLA **10** and NeoSucDOLA **15** resulted in being superior in gene transfection and were less cytotoxic than Lipofectamine, a commercially available transfection agent. Co-formulation with lipid DOPE once again increased the performance of the cholesteryl derivatives ParoChol **9** and NeoChol **14**, while it did not affect, if not decreased, the efficiencies of the dioleyl derivatives. These results can be explained considering the physico-chemical characteristics of the vectors, in particular their assembly properties. Indeed, as demonstrated by measuring the surface pressure-area isotherms, dioleyl derivatives can form monolayers at the water–air interface, probably because the presence of long alkyl chains makes the liposomes less soluble in water. However, co-assembly with DOPE likely facilitates the formation of the hydrophobic layers, resulting in better performances. Noteworthy, the presence of two AGs did not improve the efficiency of the liposome, with dimeric DiParoLinkerDiDOLA **16** as efficient as monoparomomycin derivatives. Another interesting observation coming from this study was that paromomycin derivative ParoSucDOLA **10** resulted in being more performant than the corresponding NeoSucDOLA **15** in both cell lines, even if the latter bore more amino groups of six versus four. This unexpected observation could be caused by a structural reason (the linker is anchored in different positions) or/and by the stronger DNA complexation that can lead to a lower tendency to release the cargo. Analogously to kanamycin derivatives, ParoLysSucDOLA **12** and NeoChol **14** were also able to form Chol-PEG stabilized lipoplexes when co-formulated with DOPE and to deliver DNA in vivo into mouse airways, rendering these vectors promising for further in vivo evaluations.

**Figure 8:**
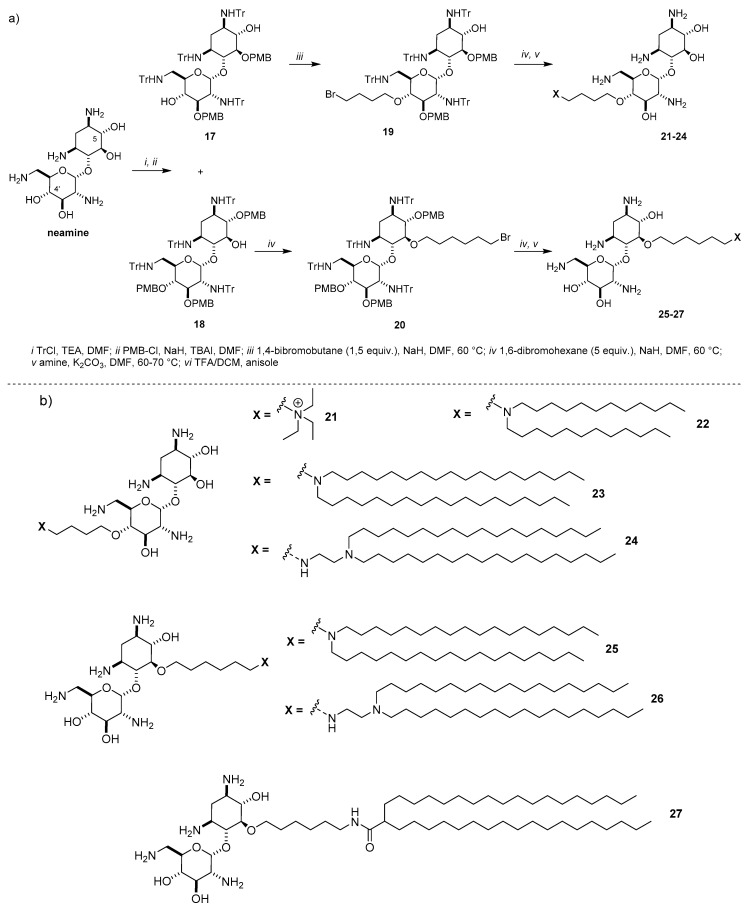
(**a**) Synthesis and (**b**) structures of lipids **21**–**27** bearing one neamine unit.

**Figure 9:**
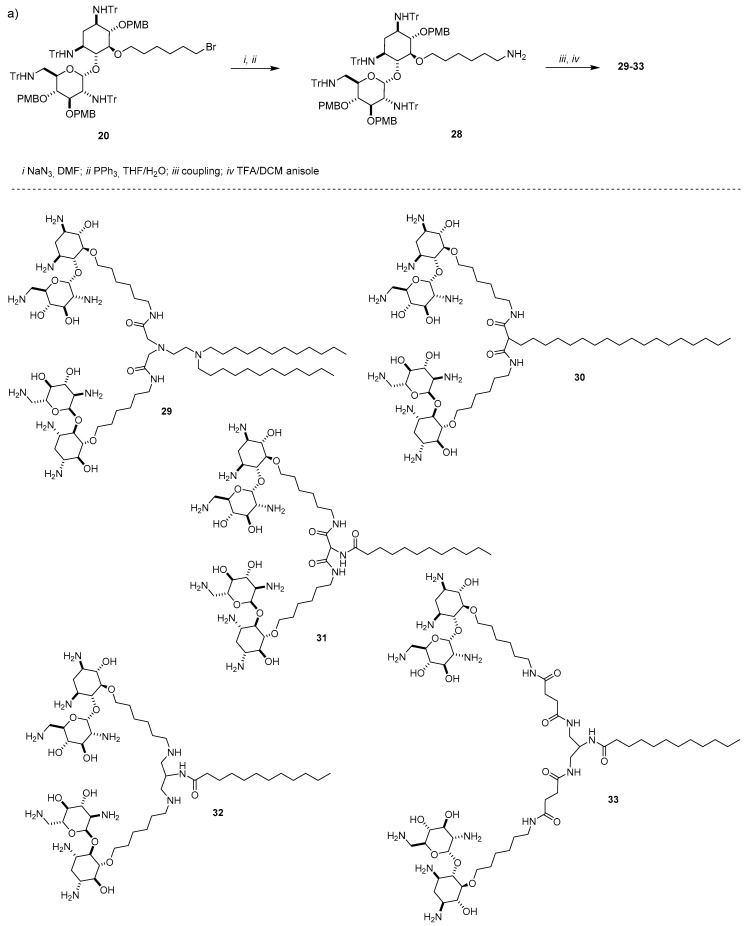
(**a**) Synthesis and (**b**) structures of lipids **29**–**33** bearing two neamine units.

The simplest AG is neamine, with 2-DOS singularly substituted at position 4 with the 6-amino-glucosaminyl group. Actually, neamine is the core scaffold for the synthesis of a selected library of cationic lipids [44]. To not sacrifice an amino function, a route to selectively functionalize hydroxyl groups at position 5 and 4′ has been developed [45]. Accordingly, after protection of the amines with the trityl group, reaction with p-metoxybenzylchloride (PMB-Cl) promoted by an excess of NaH led to the formation of a mixture of dibenzylated and tribenzylated derivatives **17** and **18**, respectively, which were separated by flash chromatography (Figure 8a). Alkylation of **17** with a slight excess of 1,4-dibromobutane produced monoalkylbromide derivative **19** in good yield and alkylation of **18** with an excess of 1,6-dibromohexane yielded intermediate **20**. Bromine of the resulting monoalkylbromides was displaced with different amines producing, after full deprotection, the target cationic lipids **21**–**26** bearing one neamine scaffold. Besides compound **21** where neamine was conjugated to an alkyl ammonium group, five neamine-based lipids were synthetized by tethering the AG to two C12 alkyl chains or two stearyl alkyl chains (C18), three of them, namely **22**–**24**, through the C4 linker at the 4′ position and two of them, **25** and **26**, through the C6 linker at position 5 (Figure 8b). A seventh derivative **27** bearing one neamine moiety and two stearyl chains was prepared by a slightly different strategy starting from bromide **20** [44]. Moreover, other five cationic lipids **29**–**33** bearing two neamine units linked to a single or double alkyl chain through different tripodal linkers (Figure 9b) were prepared by classical coupling chemistry starting from monoalkyl bromide **20**, which was converted to amine **28** by displacing the bromine atom with an azide followed by Staudinger reduction (Figure 9a).

These twelve derivatives were submitted to SARs in several cell lines giving interesting, and somewhat unexpected, insights. The results obtained, although somewhat variable within the cell line observed, can be summarized in the following points: (1) liposomes made by cationic lipids bearing only one neamine moiety were more efficient than those bearing two neamine units, those bearing two fatty acid chains performed more than those bearing only one chain; (2) stearyl chains conferred better properties to the vector compared to dodecyl alkyl chains; (3) functionalization at 4′-neamine position is preferable, probably because, being at the end of the neamine core, it facilitates the interactions with the DNA; (4) the presence of a diethylamino moiety between the linker and the fatty acid chains made the vector more performant, probably increasing the flexibility and the endosomal buffering capacity of the vector; and (5) co-assembly with DOPE facilitated the transfection ability of the vectors, as already observed in some cases with kanamycin, paromomycin, and neomycin derivatives. In general, the most performant vector resulted in being compound **24**, whose efficiency and negligible cytotoxicity, when co-formulated with DOPE, were comparable to those of Lipofectamine and ParoSucDOLA/DOPE **10**. The efficiency of compound **24** was also tested in vivo by intravenous administration of **24**/DOPE formulations in mice. However, the performances resulted in being significantly lower than those obtained with PEI 22 kDa and were similar to those obtained with ParoSucDOLA **10**/DOPE, even if no lethality was observed regardless of the doses administrated.

### 4.2. AG-Based Lipid Vectors for RNA Delivery

**Figure 10:**
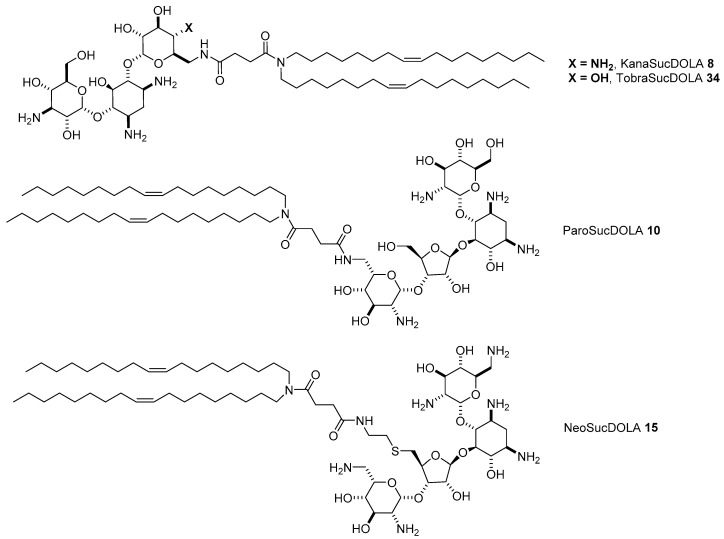
Aminoglycoside-based lipids tested as siRNA delivery vectors.

Other than DNA, RNAs can also be used as therapeutic agents for gene therapy in order to interact or produce specific functions within the cell [22]. In particular, there is great interest in the development of cationic lipids that can be exploited as efficient delivery systems for double stranded small interfering RNA (siRNA), which promotes gene silencing (protein inhibition) in the cytosol [46], and for messenger RNA (mRNA), which expresses functional proteins [47]. However, non-viral gene delivery vectors that are efficient in the delivery of DNA are not consequently efficient for siRNA or mRNA delivery as the structural characteristics of such genes quite different. Thus, soon after the application of AG-based vectors was successfully demonstrated for DNA delivery, four AG-succinyl-dioleyl derivatives, three of them KanaSucDOLA **8**, ParoSucDOLA **10**, and NeoSucDOLA **15** being the best performers in DNA delivery, along with a new tobramycin analogue, TobraSucDOLA **34**, were tested to measure their siRNA transfection efficiencies (Figure 10) [48]. 

**Figure 11:**
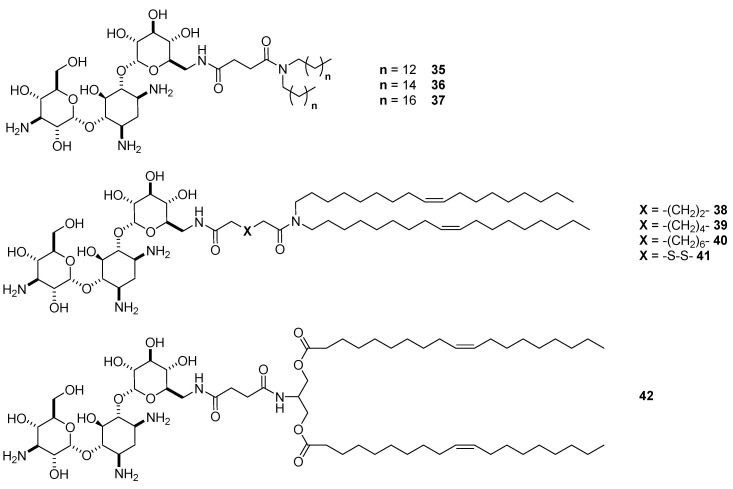
Tobramycin-based lipids **35**–**42** tested as mRNA, DNA, and siRNA delivery vectors.

The four vectors resulted in being good siRNA transfectants at various *N/P* ratios, the ability of them depending on the specific geometries. The structural features of the corresponding liposomes were determined by cryogenic transmission electron microscopy (cryo-TEM) and small-angle X-ray scattering (SAXS) experiments: 4,6-disubstituted-2-DOS lipids **8** and **34** formed liposomes having concentric multilamellar “onion like” structures exhibiting regular spacing of circa 70 Å between the consecutive repeats, with the siRNA molecules well-ordered in the lipid membrane, while 4,5-disusbituted-2-DOS derivatives **10** and **15** formed smaller liposomes with a much more disordered structure. Indeed, lipid/siRNA complexes obtained with ParoSucDOLA **10** and NeoSucDOLA **15** were more active in gene silencing in GFP-expressing d2GFP cells, probably thanks to their liposome smaller diameters (around 50 nm in positive complexes, i.e., high *N*/*P* ratios), higher colloidal stability, but, at the same time, better propensity to endosomal escape into the cytosol. In particular, the better endosomal escape likely arises from the greater flexibility of the lamellar microdomains in the siRNA complexes, favoring the so-called flip-flop mechanism [49]. Afterward, a fully SAR study was performed in order to assess the importance of the hydrophobic domains, the spacers between the cationic head and the lipophilic tails, and the behavior of stimuli responsive linkers in delivering different nucleic acids encompassing DNA, siRNA, and mRNA. Starting from tobramycin and exploiting the reactivity of the only methylenamino moiety (route A, Figure 2), a set of eight new tobramycin-based lipids was prepared (Figure 11) [50]. 

In particular, vectors **35**–**37** differentiate from the length of the lipophilic alkyl chains, C14, C16, and C18, respectively, while compounds **38**–**41** differentiate for the length of the linker (C6, C8, and C10) between tobramycin and dioleyl alkyl chains, compound **41** bearing a linear linker of six atoms with a stimuli responsive disulfide functional group in the middle. Finally, compound **42** was designed to have a similar structure to a well-known commercially available cationic lipid 1,2-dioleoyl-3-trimethylammonium-propane (DOTAP) with a hydrolizable diester function [51]. The morphologies of the liposomes made by selected candidates, namely TobraSucDOLA **34**, the conjugate with the adipic linker **38**, and the diester derivative **42** were determined by CryoTEM. In general, liposomes made with siRNA with a size between 80 and 150 nm resulted in being smaller than those obtained with mRNA and DNA, both having sizes between 200 and 500 nm. All vectors were assessed in transfection experiments in three cell lines, two of them being mammalian primary cell lines that better mimic the in vivo environment. Regardless of the nature of the gene (DNA, mRNA, or siRNA), three of the new synthetized vectors, namely the derivative with adipic linker **38**, that with palmityl linker **39**, and the diester derivative **42**, resulted in having similar, if not slightly superior transfection efficiency than TobraSucDOLA **34** in easily tranfectable HeLa cells, with compound **42** being the most efficient. Interestingly, compound **42** showed increased performance, up to 2–3 fold with mRNA, when used to transfect aorta smooth muscle cells and human fibroblast cells, being a better mRNA and DNA, and comparable siRNA vector than Lipofectamine 3000. However, the better efficiency of **42** did not seem to be correlated with the biodegradability of the ester functions, but more likely with the smaller dimension of the lipoplexes and with an easier capacity to release the nucleic acid once inside the cell. Moreover, all the vectors showed negligible cytotoxicity. On the other hand, quite surprisingly, the vector with stimuli responsive disulfide bridge **36** showed very poor transfection efficiency. Indeed, it was previously demonstrated that other amphiphilic vectors containing a disulfide bridge resulted in more efficient DNA vectors likely because the reduction of the internalized lipid facilitates the release of the cargo into the cytosol [52,53]. For liposome **41**, however, this was not the case because the reduction of the linker inhibited in some way the endosomal escape, as demonstrated by the increased efficiency observed when **41** was co-formulated with neutral fusogenic DOPE. These SAR studies confirmed that the length of the linker is an important parameter to be considered in building efficient AG-based cationic liposomes as well as the nature of the lipophilic tails, with the dioleyl chain a better choice compared to shorter or saturated alkyl chains. 

**Figure 12:**
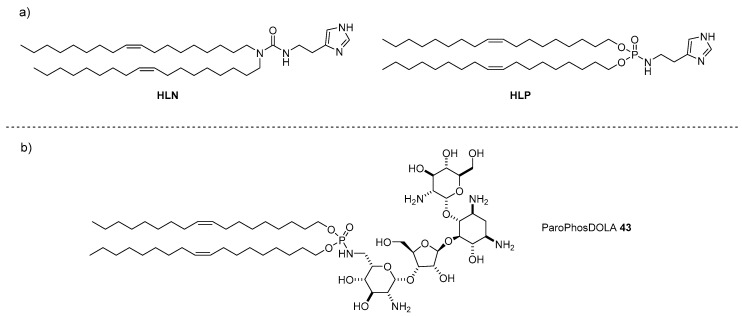
Structures of (**a**) nitrogen-based helper lipid **HLN** and phosphorous-based helper lipid **HLP**, and (**b**) ParoPhosDOLA lipid **43**.

As evidenced also for the AG-based liposomes, the efficiency of a vector to deliver the genetic material into the appropriate organelle does not depend only on the capacity to cross cell membrane. Other mechanisms such as endosomal escape and the ability to release the nucleic acid once inside the cell, among others, are very important and should be taken into consideration for the development of new universal non-viral vectors [54]. The performance of AG-based liposomes in endosomal escaping and gene complexation/decomplexation can be tuned by co-formulation with lipids, referred to as helpers, with which the lamellar phase of the lipoplexes can be modulated. In particular, the DNA transfection efficiency of ParoSucDOLA **10** when co-formulated with the lipid helpers **HLN** or **HLP** (Figure 12a), bearing a pH-buffering imidazole ring tethered to dioleyl alkyl chains through an amido- or phosphoramide-based linker, respectively, was increased most likely because the endosomal escape/DNA release mechanisms were favored [55]. Interestingly, the behavior of the two helpers was different, since the best performance was obtained when ParoSucDOLA **10** was co-formulated with **HLP**, the helper having a phosphoramide-based linker. In order to study in more depth the effect of the supramolecular assemblies in the delivery of mRNA, DNA, and siRNA, and in particular the effect of the combination of linkers **HLN** and **HLP** with paromomycin-based cationic lipids with the same lipophilic tails (dioleyl alkyl chains), ParoPhosDOLA **43** (Figure 12b) was ad hoc synthesized exploiting the same synthetic strategy seen for ParoSucDOLA **10** [56].

Four supramolecular assemblies were tested, namely homogeneous ParoSucDOLA **10/HLN** and ParoPhosDOLA **43/HLP**, where the cationic lipids and the helpers have the same amide-based or phosphoramide-based linker, respectively, and hybrid ParoSucDOLA **10/HLP** and ParoPhosDOLA **43/HLN**, where the nature of the linkers is different. Regardless of the genetic material delivered (mRNA, DNA, siRNA) and the cell lines used (HeLA, C2C12, MEF, rat aorta muscle cells, and human fibroblasts), the hybrid combinations were much more efficient than the homogeneous ones. Cryo-TEM images of the lipoplexes with DNA showed that in homogeneous assemblies, the nucleic acid is less tightly bounded because the cationic moieties are well organized in the structure, thus less available to interact with the negatively charged phosphates of the genetic material. Indeed, while hybrid liposomes showed cationic external surfaces that facilitate the interaction with the cell membrane, likely favoring internalization, homogeneous liposomes showed external corona with negatively charged DNA. This is probably the major, if not the only, reason for the different efficiencies in gene transfection between homogeneous and hybrid systems, since intracellular mechanisms such as endosomal escape and gene release in the different organelles (cytosol for RNAs and nucleus for DNA) are likely to not be affected by the different composition, with the cationic heads and the lipophilic tails identical.

**Figure 13:**
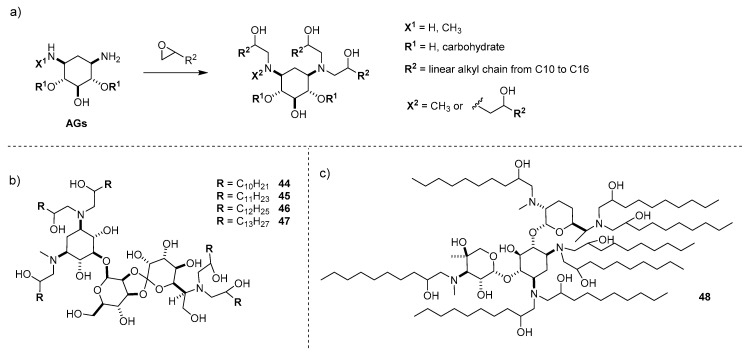
(**a**) Combinatorial synthesis of lipidic modified AGs. (**b**) Structures of the most performant lipids developed at MIT [59] and (**c**) structure of the most performant lipid developed at UT [60].

Although the aforementioned studies outlined a sort of guideline for the design of efficient AG-based gene delivery vectors, and even though it is known that efficient gene delivery vehicles could be identified through high throughput screening of libraries of liposomes [57,58], a real combinatorial approach for the SARs on AG-based cationic lipids has been exploited only in two studies, the first performed by the group of Prof. Anderson at Massachusetts Institute of Technology (MIT) and appeared in 2013 [59], and the second published this year by the group of Prof. Siegwar at University of Texas (UT) Southwestern [60]. In both studies, a one-step simple functionalization of different unprotected AGs with lipophilic epoxides and, in the second case, also with lipophilic α,β-unsaturated ester Michael acceptors, led to the preparation of libraries of AG-modified lipids that were tested for siRNA and mRNA delivery in vivo, respectively. In the first study, eight different AGs, namely neomycin, amikacin, paromomycin, ribostamycin, kanamycin, hygromycin, geneticin, and gentamicin, along with 2-DOS, were reacted with a stoichiometric amount of terminal epoxides bearing linear alkyl chain lengths of 10–16 carbons in order to fully functionalize the AG amines (Figure 13a) [59]. All the cationic lipids obtained were able to form stable nanoparticles with siRNA (up to 70% entrapment ratio) when co-formulated with cholesterol, DSPC, and PEG2000-DMG, showing a hydrodynamic diameter in the range of 60–200 nm and a slightly negative surface charge, with the PEG200 chain occupying the outer layer. The transfection efficiency of the synthetized AG cationic lipids was first evaluated in vitro by measuring knockdown of luciferase in HeLa cells using Lipofectamine 2000 as the control. Interestingly, lipids bearing aliphatic chains longer that C13 were inefficient, probably due to solubility concerns, and those with a length of C11 or C12 were the most performant. In addition, the cationic AG was found to be important, the most efficient liposomes being those bearing hygromycin, compound **45** being the best performer and **44** the second best (Figure 13b), and gentamycin (the third and fourth), probably due to their geometrical and topological features. The cationic liposomes that gave the best in vitro results were selected to study the transfection in vivo through intravenous injections in mice at 1 mg/Kg siRNA dose. The results obtained were consistent with those obtained in vitro, with hygromycin derivatives **46**, **45**, **47**, and **44**, in this order, the most performing vectors, reaching high levels (>90%) of gene silencing. Moreover, these liposomes also did not show significant toxicity at higher doses, demonstrating that they possessed all the right features to be considered between the most promising delivery vectors for in vivo gene silencing.

The success of the former study inspired the design of a second library of AG-based lipids to be tested as mRNA delivery vectors in vivo to the liver. Four different AGs, namely hygromycin, gentamycin, amikacin, and geneticin were reacted with seven epoxides bearing lipophilic tails, analogously to the former study (Figure 13a), and with five lipophilic α,β-unsaturated ester in order to fully functionalize the amino functional groups on the AG scaffold through the nucleophilic epoxide opening and Michael addition, respectively [60]. All the lipids were able to encapsulate mRNA very efficiently, forming liposomes with sizes between 100 and 200 nm, low dispersity, and a surface charge close to zero. In a preliminary in vitro screening, gentamycin- and amikacin-based liposomes gave the best results in terms of mRNA transfection on IGROV1 ovarian cancer cells when co-formulated with cholesterol, DOPE, and PEG-DMG at 20 *N/P* ratio, with those arising from the nucleophilic epoxide opening the most performant. In order to measure the in vivo delivery efficiencies and to find the best content formulations, the best candidates were administered to C57BL/6 mice through parental systemic administration by tail vain. Gentamycin derivative bearing a C10 lipophilic tail **48** (Figure 13c) resulted in being the most promising candidate to be further evaluated in future. In general, in contrast with that previously reported for siRNA delivery [59], the derivatives with gentamicin as the cationic moiety were more active than those containing hygromycin, confirming that the transfection ability of a particular vector also depends on the delivered genetic material it is designed for.

### 4.3. AG-Based Lipid Vectors with “Non-Conventional” Lipidic Tails

**Figure 14:**
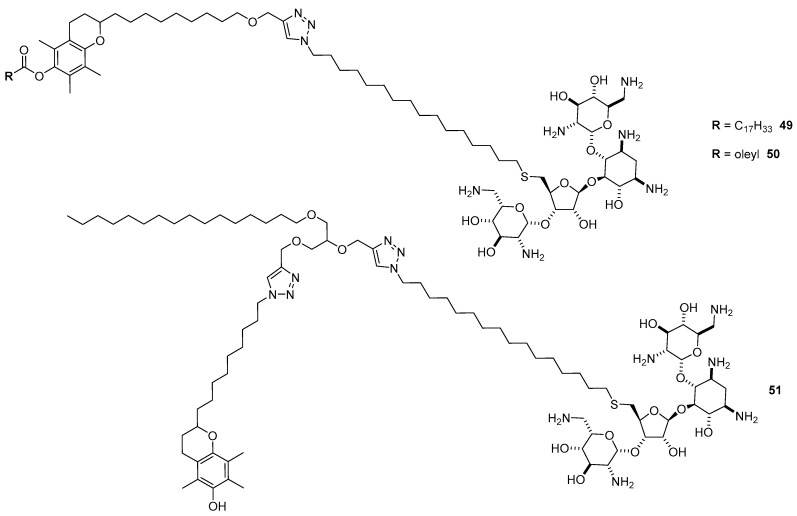
Structures of neomycin-vitamin E lipids **49**–**51**.

AGs have also been coupled to “non-conventional” lipidic tails. Three neomycin-based lipids **49**–**51** have been synthetized for efficient delivery into the liver by exploiting lipophilic vitamin E as the lipophilic tail and targeting moiety for the α-tocopherol transport protein (Figure 14) [61]. After the ability of cationic lipids to interact with the RNA duplex was assessed by UV melting analysis, Cy3-modified-siRNA was complexed with lipids **49**–**51** and the transfection efficiency of the corresponding lipoplexes was measured in mouse hepatocellular carcinoma (Hepa 1–6) cell lines. The cells treated with naked Cy3-siRNA did not show any fluorescence, while those transfected with the lipoplexes showed intense fluorescence, the liposome made with **51** being the most performant. Moreover, the effective uptake was corroborated by measuring RNAi activity in the liver cancer cells. Indeed, the liposome made with **51**, and, to a lesser extent, the liposome made with **50**, were able to induce RNA silencing in mouse LDLR-overexpressing McA-TTP cells.

**Figure 15:**
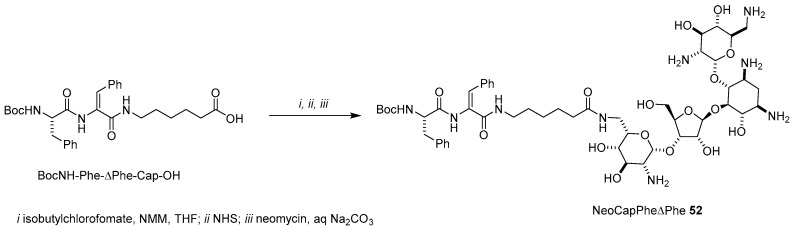
Synthesis of NeoCapPheΔPhe **52**.

AG-lipophilic peptide conjugates could be designed in order to build multifunctional liposomes with the ability to deliver genetic materials and/or lipophilic drugs into cells, maintaining, if not increasing, the ability of AGs to fight bacteria. Accordingly, *N*-Boc protected dipeptide phenylalanine-dihydrophenylalanine linked to 6-amino capronic acid (BocNH-Phe-ΔPhe-Cap-OH) was activated as a NHS-ester and reacted with unprotected neomycin yielding NeoCapPheΔPhe **52** (Figure 15) [62,63]. NeoCapPheΔPhe **52** is able to self-assemble in spherical core-shell nanostructures of around 50–70 nm with a hydrophobic core surrounded by exterior hydrophilic neomycin moieties. Such a supramolecular structure is responsible for the ability of NeoCapPheΔPhe **52** to encapsulate both hydrophobic drugs such as curcumin and eosin through hydrophobic and π–π staking interactions, and pDNA through electrostatic interactions, as evidenced by TEM (curcumin), UV fluorescence quenching (eosin), and gel retardation assay (pDNA). In particular, complete GFP-encoding pDNA complexation arises at 3.33 weight/weight (*w/w*) ratio. The transfection ability of NeoCapPheΔPhe **52** was assessed in two cell lines, namely MCF-7 and N2a, showing good efficiency to deliver GFP expressing plasmid at a 66.6 *w/w* ratio, being a better transfectant than Lipofectamine. The showed transfection efficiency mainly depends on the small size of the lipoplexes (around 40–60 nm), on the ability of the liposome to protected the plasmid from nucleases, and on the easy disassembly into the cytoplasm for nuclear localization, as demonstrated through pH studies. Besides negligible cytotoxicity, these liposomes resulted in having good antibacterial activity in both Gram-positive and Gram-negative bacterial strains.

**Figure 16:**
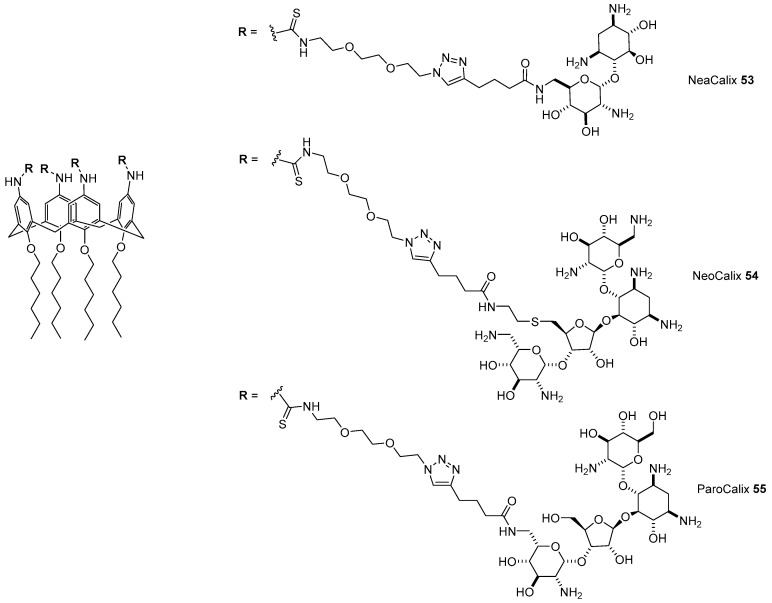
Structures of AG-calix[4]arene conjugates **53**–**55**.

Multivalency is a well-known methodology in order to amplify a molecular recognition process involving carbohydrates such as ligand/cell surface interactions [64]. One possible strategy to promote multivalency is to tether multiple ligand units to a flexible scaffold, either a molecular entity or a polymer. We thought to exploit this strategy in order to amplify the ability of AGs to interact with the cell surface, and eventually cell internalization, by tethering AGs to the upper rim of lipophilic calix[4]arenes, a macrocyclic oligomer successfully used to build efficient gene delivery vectors by anchoring guanidinylated moieties [65,66]. Accordingly, after functionalization of neamine, paromomycin, and neomycin with a linker bearing an isothiocyanate moiety, we used tetraminocalix[4]arene to build three AGs-calix[4]arene cationic lipids, namely NeaCalix **53**, NeoCalix **54**, and ParoCalix **55**, through isothiocyanate-amine click chemistry (Figure 16) [67]. The ability to complex pDNA encoding modified firefly luciferase was assessed through the fluorophore-exclusion titration assay showing that AGs-Calix **53**–**55** were able to completely pack DNA around the 1.5–2 *N/P* ratio, exhibiting greater DNA complexation ability than the gold standard transfectant bPEI 25 kDA. Moreover, having at their optimal *N/P* ratio a hydrodynamic diameter around 150 nm with an overall positive surface charge, these lipoplexes displayed suitable features for gene delivery. Indeed, AGs-Calix **53**–**55** at their optimal *N/P* charge ratios exhibited transfection efficiencies in two cell lines higher than those of the gold standard bPEI, with ParoCalix **55** as the best performant, even in the presence of serum, which better mimics the in vivo environment, showing at the same time negligible cytotoxicity. Remarkably, AGs-Calix scaffolds **53**–**55** and their lipoplexes with pDNA were able to inhibit Gram-negative *E. coli*. growth, with the lipoplexes being more effective. Moreover, it is known that AGs are most effective against Gram-negative bacteria, AGs-Calix **53**–**55**, and their pDNA complexes also showed good antibacterial activity against Gram-positive *S. lutea*, even if to a lesser extent.

## 5. Polymeric AG-Based Gene Delivery Vectors

Along with cationic liposomes, natural and synthetic polymers have been widely studied in order to seek non-viral vectors able to surmount all the physiological barriers for an ideal gene delivery vector [68,69,70]. Cationic polymers can be basically grouped in two classes: cationic polymers that are commercially available, encompassing gold standard bPEI, lPEI, poly-lysins, PAMAM dendrimers, and chitosan, which are probably the most studied as they are easy to functionalize, and the cationic polymers that are ad hoc designed and synthetized. In this section, we will outline the chemical and biological properties of polymers bearing AGs, either introduced to functionalize commercially available cationic polymers or used as building blocks to build new hyperbranched structures.

### 5.1. Functionalization of Polymers with AGs

**Figure 17:**
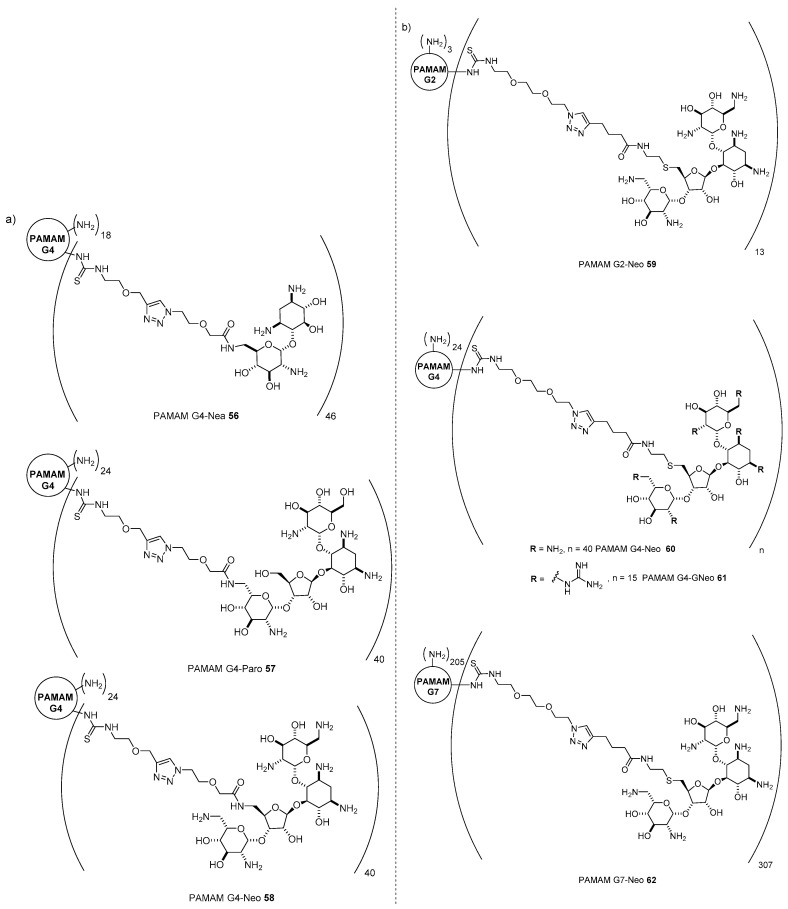
(**a**) Structures of AG-based PAMAM G4 conjugates **56**–**58** and (**b**) structures of neomycin based PAMAM dendrimers of different generations **59**–**62**.

PAMAM dendrimers are certainly among the most studied cationic polymers in gene delivery [71]. Indeed, they possess several features that render their use very intriguing such as well-defined architecture, easy functionalization, the presence of tertiary amines able to act as proton sponges, and negligible cytotoxicity. However, undecorated PAMAM dendrimers, besides the generation considered, are not very efficient, being less efficient than the gold standard bPEI. For this reason, in order to increase their efficiencies while maintaining their negligible cytotoxicity, a lot of interest has been paid to the functionalization of the outer shell of PAMAM dendrimers with different moieties, ranking from cationic, anionic, and lipophilic to targeting moieties [30]. In this scenario, we decided to assess the potential of AG-based PAMAM vectors by linking three aminoglycosides, namely neamine, neomycin, and paromomycin, to PAMAM generation four (PAMAM G4), which is the most studied PAMAM dendrimer showing the best balance between efficiency and toxicity [72]. In order to exploit isothiocyanate-amine click chemistry, neamine, paromomycin, and neomycin were functionalized with a linker bearing the suitable isothiocyanate group through the synthetic pathways depicted in Figure 2 of path A for neamine and paromomycin, and path B for neomycin, and successively clicked to the dendrimer outer primary amines. Due to steric constraints, full functionalization of the 64 outer PAMAM G4 amines was not possible and PAMAM G4-Nea **56**, PAMAM G4-Paro **57**, and PAMAM G4-Neo **58** bearing 46, 40, and 40 AGs, respectively, were obtained, as assessed by ^1^H-NMR and MALDI spectroscopy (Figure 17a). Interestingly, conjugates **56**–**58** were less able to complex DNA than undecorated PAMAM G4. However, they were able to fully complex pDNA at a low *N/P* ratio and form poliplexes with a hydrodinamic diameter between 150 and 200 nm. The transfection efficiency of poliplexes prepared with luciferase expression plasmid pGL3 at different *N*/*P* ratios was assessed in three cell lines, namely HeLa, Cos-7, and U87, in complete medium and in the presence of increasingly high concentration of serum. As expected, it was not possible to find an optimum value of *N/P* ratios for all the poliplexes, but PAMAM G4-Paro **57** and PAMAM G4-Neo **58** showed better transfection efficiencies and lower cytotoxicity than gold standard 25 KDa bPEI (and much better than undecorated PAMAM G4), each one at their optimal *N/P* ratio. In particular, PAMAM G4-Paro **57** resulted in being more performant than bPEI, even in the presence of 10% and 50% in volume of fetal bovine serum (FBS). Afterward, we decided to also investigate the role of the PAMAM generation [73]. Accordingly, with the same synthetic strategy and a slightly different linker, we prepared four new conjugates starting from PAMAM dendrimers of different generations, namely PAMAM G2-Neo **59**, PAMAM G4-Neo **60**, and PAMAM G7-Neo **62**, bearing 13, 40, and 307 neomycin moieties, respectively (Figure 17b) [73]. Moreover, in order to provide further insights on the different behavior of AGs and guanidinoglycosides in gene delivery, we also prepared PAMAM G4-GNeo **61**, to which only 15 guanidinoneomycins were possible to tether due to the higher steric hindrance of the guanidine group compared to the amino function. Interestingly, conversely to PAMAM with higher generations, the ability to complex DNA of PAMAM G2 was increased by conjugation with neomycin. Nevertheless, besides PAMAM G7, the transfection efficiencies of undecorated PAMAM dendrimers were raised by conjugation with neomycin at almost any *N/P* ratios, as assessed by treating two different cell lines, namely HeLa and COS-7 cells, with pGL3 poliplexes, which were more performant than the gold standard bPEI 25 KDa. Interestingly, PAMAM G2-Neo **59** resulted in being the most performant vector for different values of *N/P* ratios, maintaining in all cases negligible cytotoxicity. This result is noteworthy because the production of PAMAM G2, which in general is less efficient in delivering drugs and less cytotoxic compared to PAMAM G4, is much less costly and time consuming. Moreover, PAMAM G2-Neo **59** showed very good antibacterial activity against *E. coli*. in a concentration-dependent manner. As already demonstrated with AG-based liposomes, guanidinylation of the AG amines did not favor the overall activity of the vector, with PAMAM G4-GNeo **61** being less efficient than both PAMAM G2-Neo **59** and PAMAM G4-Neo **60**.

**Figure 18:**
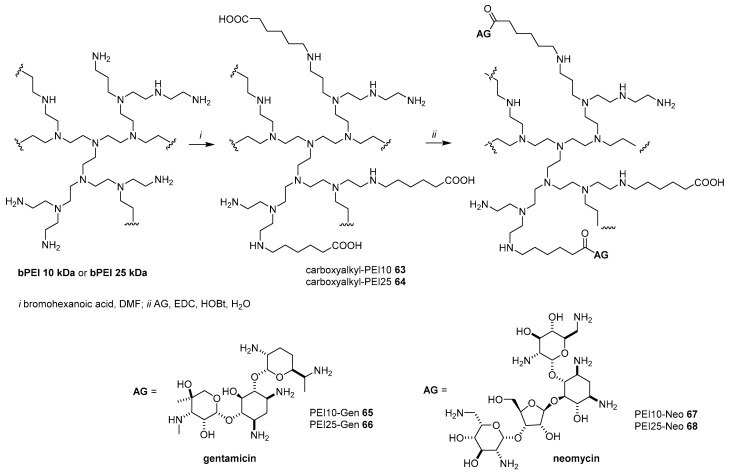
Synthesis of AG-PEI conjugates **65**–**68.**

In general, polymer functionalization is intended not only to increase the transfection efficiency of the original polymer, but also, in some cases, to render the vector selective by tethering targeting moieties [30]. In this sense, bPEI 10 KDa and 25 KDa, which are their self-efficient polymeric non-viral vectors, have been functionalized with gentamycin and neomycin in order to enhance the transfection efficiency in the kidney by targeting megalin transmembrane protein [74]. Indeed, it has been demonstrated that megalin is a highly expressed receptor in proximal tubular epithelial cells, which are the most populous cell type in the kidney. Along with cubilin protein, megalin is involved in the endocytic uptake of many ligands and in the retention of drugs including AGs [75]. Accordingly, bPEI 10 kDa and 25 kDa were reacted with different quantities of 6-bromohexanoic acid (around 10%, 30%, and 50% primary amine substitution) in order to obtain carboxyalkyl-PEI10 and -PEI25 **63**–**64**, respectively, having a different degree of functionalization (Figure 18). The resulting polymers bearing free carboxylic acid functions were coupled to unprotected gentamicin and neomycin AGs, leading to the formation of PEI10-Gen, PEI25-Gen, PEI10-Neo, and PEI25-Neo **65**–**68**, respectively, with different degrees of grafting.

Interestingly, PEI-AG conjugates formed smaller polyplexes compared to undecorated PEIs: at *C/P* = 2, PEI25-AGs **66** and **68** formed polyplexes with an average size of about 143–163 nm, while PEI10-AGs **65** and **67** had an average size of about 166–170 nm. The transfection efficiencies of polyplexes formed by complexation of pDNA encoding EGFP with AG-based polymeric vectors **65**–**68** were tested in megalin-expressing cell lines HK-2 and MDCK and the non-megalin-expressing HepG2 cell line as a comparison. Notably, AG-PEI vectors were able to transfect megalin-expressing cell lines with higher efficiency than undecorated PEIs and carboxyalkyl-PEIs **63**–**64** at *C/P* ratios higher than 2 (around 7-fold increase), the performance of the vectors being dependent from the PEIs’ molecular weights (the higher, the better), the AG grafted, and the degree of grafting. In general, gentamicin enhanced the performance of the vector to a greater extent than neomycin, where the higher the degree of grafting, the higher the transfection efficiency. The most performant polyplex was PEI25-Gent **66** with around 10% of gentamicin grafted (calculated on the original PEI free amines) when formulated at *C/P* = 4. More interestingly, regardless of the *C/P* ratio, a much higher transfection efficiency was observed in megalin-expressing cell lines compared with non-megalin expressing cell lines with the increase of AGs moieties tethered to the vector, reaching a 7-fold increase for the best cationic polymer **66**. Moreover, megalin specificity was corroborated by performing transfection studies in the megalin-expressing NK-2 cell line in the presence of increasing quantities of human serum albumin, a known targeting megalin competitor. Accordingly, the transfection efficiency of vectors **66** and **68** was inhibited with the increase of albumin, where complete inhibition was reached with a dose of 20 mg/mL.

### 5.2. Synthesis of Hyperbranched Polymers Based on AG Scaffolds

**Figure 19:**
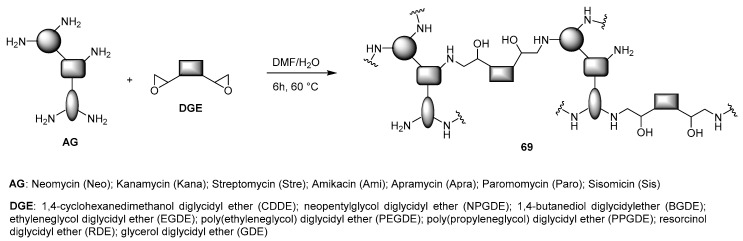
Synthesis of AG-DGE hyperbranched polymers **69**.

The paradox according to which the transfection efficiency and cytotoxicity of a cationic polymer are often proportional is probably one of the most important issues to be challenged in the development of new polymeric gene delivery vectors [28]. Accordingly, a possible alternative of commercially available polymeric transfectants such as PEI and PAMAM dendrimers is the synthesis of novel hyperbranched polymers starting from natural, biodegradable building blocks. AGs, being natural compounds bearing many functional groups, and in particular primary amines, can be exploited for the preparation of hyperbranched polymeric structures with different cross-linkers. The combination of combinatorial synthesis, high throughput screening, and chemoinformatic models in order to predict which are the key physicochemical parameters to take into account is often a winning strategy to find lead candidates, as frequently demonstrated in medicinal chemistry [76]. Accordingly, the group of Prof. Rage has developed a quantitative structure–activity relationship (QSAR) modeling for correlating the properties of AG-based hyperbranched polymers with their ability to deliver pDNA into the cell. In a pioneering study, a library of 56 AG-based hyperbranched polymers **69** was synthesized through epoxide ring opening polymerization starting from seven AGs and eight diglycidyl ethers (DGE) (Figure 19) [77].

**Figure 20:**
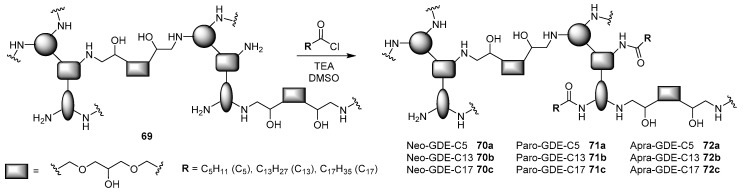
Synthesis of AG-GDE lipophilic polymers **70**–**72**.

A first screening was performed in two cancer cell lines, namely PC3 human prostate cells and Mia PaCa-2 pancreatic cancer cells, with polyplexes formed at 25:1 polymer/pGL3 plasmid weight ratio. The seven most performant candidates, Apra-RDE, Paro-RDE, Paro-GDE, Sis-RDE, Sis-GDE, Ami-EGDE, and Neo-GDE were selected for further physicochemical characterizations and experiments. Their transfection efficiencies were evaluated independently with two plasmids (pGL3 and pEGFP) at different *N/P* ratios and correlated with the gold standard bPEI 25 kDa. For most of the polymers, the best performances were observed between 50 and 100 *N/P* ratio, with the AG-based polymers being more efficient than the bPEI 25 kDa, probably thanks to their reduced cytotoxicity. Physicochemical parameters of the polymers are very important in promoting efficient transgene expression and were used to develop QSAR models with support vector regression (SVR). Exploiting five different descriptor series, the authors were able to build cheminformatic modeling, which resulted in being highly predictable in a new set of experiments. Since one of the major contributions for the efficient pDNA delivery of AG-DGE polymers was an enhanced hydrophobic character, the same group was speculated to further increase their transfection ability by functionalization with lipophilic chains. Accordingly, three of the most performing polymers, namely Neo-GDE, Paro-GDE, and Apra-GDE, were reacted with three fatty acid acyl chlorides with respectively six-, fourteen-, and eighteen-linear carbon length chains in three different molar ratios, producing a library of 27 lipopolymer candidates (Figure 20) [78].

After some physicochemical properties were successfully assessed such as pDNA complexation, hydrodynamic diameter determination, and zeta-potential measurement, which showed that lipidic polymers **70**–**72** possessed the suitable requirements for a polymeric vector, the transfection efficiencies in different cell lines, without and in the presence of up to 50% of serum, were measured at different *N/P* ratios. The results obtained, and corroborated by chemoinformatic modeling, showed that the presence, in low percentage, of long stearyl chains was fundamental in order to increase the delivery performance of the polymers, being Neo-GDE-C17 **70c** and Apra-GDE-C17 **72c** obtained by mixing acyl chloride/polymer in 1:2 molar ratio, where the most performant vectors displayed higher transgene expression than both bPEI 25 kDa and the undecorated polymer, even in the presence of serum.

**Figure 21:**
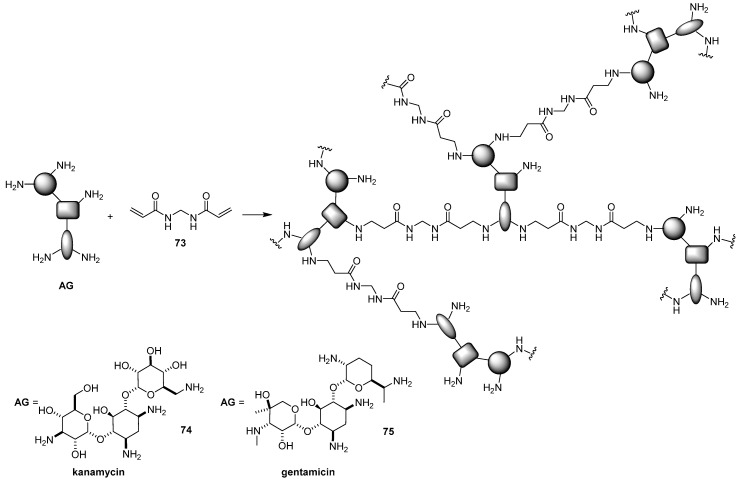
Synthesis of kanamycin- and gentamicin-based hyperbranched polymers **74**–**75**.

In addition to constructing new biodegradable polymers, there is a general increasing interest in exploiting stimuli responsive linkers [54]. Accordingly, using Michael acceptor N,N’-bismethylenebisacrylamide **73** as degradable linkers, two AG-based hyperbranched polymers **74**–**75** were prepared starting from unprotected kanamycin and gentamicin, respectively (Figure 21) [79,80]. The formed polymers showed several features suitable for efficient gene delivery vectors such as great ability to fully complex pDNA at very low *N/P* ratios, polyplex dimensions lower than 100 nm, degradation in acidic medium, and high buffering capacity, which facilitates endosomal escape and negligible cytotoxicity. The delivery performances of poliplexes formed by **74**–**75** and pDNA encoding EGFP evaluated in HeLa and COS-7 cell lines at different *N/P* ratios showed an increase in the transfection efficiencies with an increase in the *N/P* ratios, reaching the plateau at *N/P* = 50, which was much more active than the naturally occurring chitosan polysaccharide and approached the efficiency of 25 kDa bPEI. Interestingly, the performance of polymer **75** was only slightly affected by the presence of serum, rendering such a polymer as a promising gene delivery vector to be tested in vivo. Moreover, the same gentamicin-based branched polymer **75** displayed good antitumor and antibacterial activities, as assessed by the MTT assay against HeLa cancer cells and by measuring the inhibition ratio against *E. coli*, respectively [80].

## 6. Conclusions

AGs have been intensively used to fight bacteria over eight decades, since streptomycin was discovered in the 1940s. However, the development of antibacterial resistance, principally due to modification of the AG scaffold by AG-modifying enzymes as well as the frequently occurring toxic side effects such as ototoxicity and nephrotoxicity, could have led to the end of this class of antimicrobials. However, if we consider the scientific productivity with the word “aminoglycoside(s)” in the title, we notice that more than one thousand papers has appeared in the literature (Web of Science source) in the last five years, meaning that AGs are still of great appeal to the scientific community. The continuing (and somewhat growing) interest in AGs arises from two main accomplishments: (1) a better knowledge of the chemistry of AGs, which provides the scientist with a rather wide portfolio of possibilities, and (2) a better understanding of the AG mechanisms of action, which leads to a broader range of applications. Among the new trends, the use of AGs as cationic moieties to build multifunctional non-viral gene delivery vectors has attracted the attention of the scientific community working in the field. Different libraries of cationic lipids have been produced by tethering lipophilic tails to AG cationic heads through suitable linkers, encompassing stimuli responsive linkers, and using them to promote the delivery of different genetic materials such as DNA, mRNA, and siRNA to the target organelles. Although great effort has been made in this area, an ideal AG-based lipidic vector is not available yet. However, these studies shed light on some important insights: (1) it is very important to maintain a certain hydrophilicity/lipophilicity balance; (2) the AGs charge density and geometry have great influence on the vector performance; (3) the design of AG-based vectors bearing two long alkyl chains (stearyl or oleyl) is often a winning choice; (4) the use of linkers that are not too short is preferable; and (5) although guanidinoglycosides are known to be stronger RNA binders and to facilitate internalization of bio-macromolecules, guanidination of AG-based gene delivery liposomes does not contribute to better transfection efficiency. Along with AG-based liposomes, AGs have also been exploited to synthesize cationic polymeric non-viral vectors, both by functionalizing pre-existing commercially available polymers such as bPEI and PAMAM dendrimers, and by using their selves as scaffolds in polymerization reactions. In the former cases, more performant polymers than the gold standard bPEI 25 kDa were obtained at their optimal *N/P* ratio with neomycin and paromomycin, the cytotoxicity being similar or lower. Wide libraries of AG-based polymers were prepared starting from AG scaffolds and Michael acceptor bis-acrylamides or terminal bis-epoxide linkers, and with the help of ad hoc developed QSAR modeling; in this case, very efficient vectors have also been found, showing promising potential for future applications. However, even if a general rule of thumb can be extrapolated, it is still very difficult to be able to foresee the behavior of an AG-based polymer, since multiple barriers in the gene delivery process should be taken into consideration.

Most of the results presented in this review come from in vitro experiments carried out to measure the transfection efficiencies of AG-based liposomes and polymers in different cell lines, some of them in the presence of serum, which better mimics the in vivo environment. A few in vivo experiments have been performed through intranasal instillation of Chol/PEG stabilized DOPE/lipoplexes to BALB/c mice or parenteral systemic administrations of Chol/PEG/DOPE stabilized lipid-modified AGs to C57BL/6 mice, showing promising preliminary results to be pushed forward for the design of multimodular AG-based vectors for in vivo gene delivery.

In general, although some AG-based vectors are efficient transfectants, there are still challenges to overcome in order to be able to find the “ideal” vector that would be able to approach the efficiency of some viral vectors. However, thanks to the renaissance that AGs are facing, we believe that the community should stay optimistic in this sense.
